# Amyloid‐β protein and MicroRNA‐384 in NCAM‐Labeled exosomes from peripheral blood are potential diagnostic markers for Alzheimer's disease

**DOI:** 10.1111/cns.13846

**Published:** 2022-04-26

**Authors:** Ying Li, Shuang Meng, Wu Di, Ming Xia, Lei Dong, Yue Zhao, Sihai Ling, Jing He, Xiaoxing Xue, Xiali Chen, Chengeng Liu

**Affiliations:** ^1^ Clinical Laboratory of Beijing Anding Hospital Capital Medical University Beijing China; ^2^ Clinical Laboratory of Air Force General Hospital Chinese People's Liberation Army Beijing China; ^3^ 96698 State Key Laboratory for Disease Prevention and Control National Institute for Communicable Disease Control and Prevention Beijing China; ^4^ Clinical Laboratory of Xuanwu Hospital Capital Medical University Beijing China; ^5^ Clinical Laboratory of Minhang Hospital Fudan University Shanghai China

**Keywords:** Alzheimer's disease, ATP‐binding cassette transporter A1, biomarker, exosome, neural cell adhesion molecule

## Abstract

**Objective:**

We aimed to establish a method to determine whether amyloid‐β (Aβ) protein and miR‐384 in peripheral blood neural cell adhesion molecule (NCAM)/ATP‐binding cassette transporter A1 (ABCA1) dual‐labeled exosomes may serve as diagnostic markers for the diagnosis of Alzheimer's disease (AD).

**Methods:**

This was a multicenter study using a two‐stage design. The subjects included 45 subjective cognitive decline (SCD) patients, 50 amnesic mild cognitive impairment (aMCI) patients, 40 AD patients, and 30 controls in the discovery stage. The results were validated in the verification stage in 47 SCD patients, 45 aMCI patients, 45 AD patients, and 30 controls. NCAM single‐labeled and NCAM/ABCA1 double‐labeled exosomes in the peripheral blood were captured and detected by immunoassay.

**Results:**

The Aβ42, Aβ_42/40_, Tau, P‐T181‐tau, and miR‐384 levels in NCAM single‐labeled and NCAM/ABCA1 double‐labeled exosomes of the aMCI and AD groups were significantly higher than those of the SCD, control, and vascular dementia (VaD) groups (all *p* < 0.05). The Aβ42 and miR‐384 levels in NCAM/ABCA1 dual‐labeled exosomes of the aMCI and AD groups were higher than those of the control and VaD groups (all *p* < 0.05). The exosomal Aβ42, Aβ_42/40_, Tau, P‐T181‐tau, and miR‐384 levels in peripheral blood were correlated with those in cerebrospinal fluid (all *p* < 0.05).

**Conclusion:**

This study, for the first time, established a method that sorts specific surface marker exosomes using a two‐step immune capture technology. The plasma NCAM/ABCA1 dual‐labeled exosomal Aβ_42/40_ and miR‐384 had potential advantages in the diagnosis of SCD.

## Graphical abstract

In this study, we applied a newly developed technology to capture peripheral blood exosomes with both NCAM and ABCA1 protein tags. We found that Aβ42 and miR‐384 in these exosomes have great potential value for the diagnosis of SCD and aMCI.

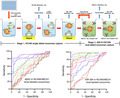

## 1 INTRODUCTION

Diagnosis of Alzheimer's disease (AD) requires physical signs, psychological tests, syndrome judgment, and biomarker testing. AD biomarkers can generally be divided into two categories: imaging markers and clinical laboratory diagnostic markers. In vivo diagnostic imaging markers such as amyloid positron emission computed tomography (PET) imaging have good diagnostic specificity on AD and also have potential value in the diagnosis of subjective cognitive decline (SCD). However, the expensive cost of PET limits its application as a routine inspection approach.^1^, ^2^ At present, there are no drugs that can effectively treat patients with Alzheimer's‐type dementia (DAT), but patients in the middle and early stages such as amnestic mild cognitive impairment (aMCI) and SCD could avoid or delay the development of the disease through a series of treatments.^3^, ^4^ Therefore, it is important to make a clear diagnosis in the early stages when amyloid β‐protein (Aβ) has not yet significantly accumulated.^5^ Recent studies have shown that the increase and aggregation of Aβ outside the cells may not be the earliest molecular changes in AD. A series of molecular biological changes have begun to occur in neuronal cells before the occurrence of Aβ aggregation that can be detected by PET, which is considered the earliest (ultra‐early) change that leads to the occurrence, progression, and clinical onset of AD.^5^, ^6^, ^7^ In recent years, with the conclusion of a number of large‐scale long‐term longitudinal studies, the clinical application value of multiple new familial AD ultra‐early diagnostic markers has been initially clarified. For example, molecules such as peripheral blood neurofilament light chain (NfL) are expected to provide early warning of the onset of familial AD with presenilin 1 mutant 10–15 years in advance, to achieve ultra‐early early warning.^8^ However, for sporadic AD, which accounts for 90% of the total AD cases, there is still a lack of effective peripheral blood ultra‐early warning biomarkers.

The cerebrospinal fluid (CSF) is in direct contact with the brain, and the material exchange with the brain is more direct than that of peripheral blood or other body fluids. Therefore, CSF is considered a clinical laboratory diagnostic biomarker specimen that can diagnose AD and other neurological diseases more specifically and earlier.^2^ However, because CSF needs to be collected invasively by lumbar puncture, there is a certain clinical risk and low patient acceptance. Thus, it is difficult to carry out CSF examination routinely and on a large scale. Due to the existence of the blood–brain barrier (BBB) transport mechanism, CSF, and peripheral blood can also exchange certain substances. In recent years, a series of studies have suggested the possibility of using peripheral blood as a specimen source for AD diagnostic markers. In addition, peripheral blood collection is less traumatic, and patient acceptance is high. If markers with high diagnostic efficiency can be found, it will greatly promote the AD marker routine detection and effectively increase the AD detection rate. However, some potential AD markers, such as proteins and microRNAs (miRs), that have great clinical value in CSF, show unsatisfactory diagnostic performance when directly detected in peripheral blood. The reason may be that the BBB transport process leads to changes in the quality or quantity of the markers or the interference caused by the secretion of the same or similar substances in other tissues and organs.^2^, ^9^

At present, more studies focus on noninvasive approaches for AD early diagnosis or warning. The protein biomarker panels, metabolomics biomarker panels, and nucleic acid biomarker panels have been clarified.^10^ miRs are a class of small (18–25 nucleotides), single‐stranded noncoding RNAs involved in the posttranscriptional regulation of gene expression. In recent years, miRs have been shown to play important roles in several diseases, such as cancer, cardiovascular disease, diabetes, and central nervous system diseases. Because of their stable characteristics, altered miRs in tissues and organs may lead to the dysregulation of miRs in body fluids, such as CSF, serum, and urine, either by cell destruction or secretion. Therefore, miRs are attractive targets in the search for novel biomarkers.^10^, ^11^ Given the complexity of miR sources, how to detect miRs that represent AD pathological changes has become one of the solutions to reduce false negatives in diagnosis.

In a previous study, we have reported that miR‐384 could downregulate the expressions of amyloid precursor protein (APP) and beta‐secretase‐1 (BACE‐1), as well as the activity of BACE‐1.^12^ Further animal experiments have shown that miR‐384 was downregulated in hippocampal neurons of APP/presenilin 1 (PS1) double transgenic mice. At the same time, the total miR‐384 in CSF significantly increased, suggesting that the reduced miR‐384 in neuronal cells may be released into CSF. However, the total miR‐384 in mouse plasma did not change significantly, while the plasma exosomal miR‐384 significantly increased, compared with that in the wild‐type mice. The above phenomenon has also been found in AD patients.^13^ These results suggest that miR‐384 in CSF is transported to peripheral blood through exosomes across the BBB. Further exosome protein mass spectrometry results have shown that the expression of ATP‐binding cassette transporter A1 (ABCA1) in the CSF of AD patients was significantly higher than that of controls, suggesting that ABCA1 may be related to the increase of peripheral blood exosomal miR‐384.

Previous multicenter studies have shown that the concentration of neural‐derived exosomal Aβ42, T‐tau, and P‐T181‐tau in peripheral blood can reflect their changes in CSF.^14^ In the present study, the neural cell adhesion molecule (NCAM) single‐labeled exosomes were first captured. Then, specific NCAM/ABCA1 dual‐labeled exosomal components were screened for corresponding detection. All subjects were tested for NCAM single‐labeled and NCAM/ABCA1 dual‐labeled exosomal Aβ42, Aβ_42/40_, T‐tau, P‐T181‐tau, NfL, and miR‐384. The results from the discovery stage were validated in a validation stage with more samples from different centers.

## 2 METHODS AND MATERIALS

### 2.1 Participants

A total of 392 subjects were randomly selected from the Beijing Anding Hospital (center 1), Xuanwu Hospital of Capital Medical University (center 2), Minhang Hospital, Fudan University (center 3), and Air Force General Hospital, Chinese People's Liberation (center 4) from May 2018 to March 2021, including 92 subjects in the SCD group, 95 subjects in the MCI group, and 85 subjects in the DAT group. Another 60 randomly selected healthy subjects were recruited as the control group. To evaluate the differential diagnosis, 60 patients with vascular dementia were also included. The diagnosis of AD was made according to the criteria of the National Institute on Aging and Alzheimer's Association (NIA‐AA).^15^ The diagnosis of aMCI was based on published criteria.^16^ The diagnosis of SCD was made according to published criteria and research.^17^, ^18^ The diagnosis of VaD subject made according to published criteria.^19^, ^20^ Written informed consents were obtained from all participants or their legal guardians. This study was approved by the Institutional Review Board of AnDing Hospital and Xuanwu Hospital, Capital Medical University.

The patients enrolled in three central hospitals in Beijing participated in the discovery stage of the study; the patients enrolled in the two central hospitals in Shanghai and Heilongjiang participated in the verification stage. Only the patients without major psychiatric or neurologic disorders, alcoholism, renal or hepatic disease, diabetes mellitus, hyperthyroidism, chronic obstructive pulmonary disease, or unstable cardiac disease were enrolled in this study. All samples were stored and detected in a blinded manner.

### 2.2 Sample collection and pretreatment

The EDTA anticoagulated venous blood was drawn in the morning after 8–12 h fasting (Vacutainer K_2_‐EDTA tube, BD, Franklin Lake,). To reduce the error caused by the exchange of exosomes and blood, centrifugation was performed within 30 min after specimen collection. Two tubes of specimens were collected from the subjects undergoing blood cell exosomes release experiments, and one tube of specimens was collected from other subjects. To obtain plasma, the specimens were centrifuged at 4200 *g* for 10 min at 4°C. To obtain blood cells, the blood separation medium was used according to the manufacturer's protocol (Solarbio).^14^ The plasma was stored in liquid nitrogen before testing and was only allowed to freeze and thaw once. The blood cells were used in the following experiment within 1 h.

The CSF samples were collected within 1 h after blood samples collection following the CSF collection and biobanking guideline.^21^ In brief, the patient was positioned in a left lateral position, and the puncture point was between the waist 3 and 4. A total of 5–15 ml of CSF was collected from each subject and centrifuged at 2000 *g* at 4°C for 10 min.^13^, ^14^ The supernatant was immediately collected into a polypropylene tube, stored in liquid nitrogen before testing, and allowed to freeze and thaw only once.

### 2.3 Blood cell separation and culture

The separated red blood cells (RBCs) and white blood cells (WBCs) were washed in 37 °C pre‐warmed phosphate‐buffered saline (PBS) three times and then cultured in RPMI 1640 medium (Invitrogen,) containing 10% exosome‐depleted serum (Gibco, Thermo Fisher Scientific,) at a concentration of 1 × 10^7^/L for 0 h and 2 h. The NCAM in the cells and medium were detected using an enzyme linked immunosorbent assay (ELISA) kit (Abcam,) according to the manufacturer's protocol. The randomly selected and gender/age‐matched SCD, aMCI, DAT, VaD, and control samples from Xuanwu Hospital were run in triplicate.

### 2.4 Collection of neuronal‐derived exosomes from plasma

The total exosomes were extracted from plasma. In brief, 0.5 ml plasma was incubated with 0.15 ml thromboplastin‐D (Amresco,) for 60 min at room temperature. Then, 0.33 ml magnesium and calcium‐free Dulbecco's phosphate‐buffered saline (D‐PBS, Amresco), 0.02 ml EDTA‐free protease, and phosphatase inhibitor cocktails (Abcam) were added. Next, 0.5 ml of the mixture was added to 0.5 ml D‐PBS. The mixture was centrifuged at 1500 *g* for 20 min at 4°C. The supernatant was mixed with an equal volume of D‐PBS. The mixture was centrifuged at 1500 *g* for 20 min.

Then, specific neuronal‐derived exosomes defined by multiple studies were separated using a novel immunomagnetic bead method.^14^, ^22^ In brief, 0.2 ml supernatant of the previous step was mixed with 0.05 ml ExoQuick^TM^ exosome precipitation solution (System Biosciences) and incubated on ice for 1 h. After centrifugation at 1500 *g* for 30 min, the pellet was resuspended in 200 μl D‐PBS. Each sample (200 μl) was incubated with the NCAM monoclonal antibody (mouse antihuman, Abcam)‐coated Dynabeads (1 μm, Thermo Fisher Scientific) for 30 min in a microplate on a shaker at 37°C. Then, a magnet that fits the microplate was used to capture the magnetic beads, and the supernatant was discarded. After removing the magnet, each sample was resuspended in 210 μl 0.05 M glycine–HCl (pH 3.0, Amresco) and mixed with 0.10% Tween 20 and 10 μl inhibitor cocktails by shaking for 30 s, followed by an additional magnet capture procedure. The supernatant was harvested, and the pH was adjusted to 7.0 with 1 M Tris–HCl (pH 8.6, Amresco).

**FIGURE 1:**
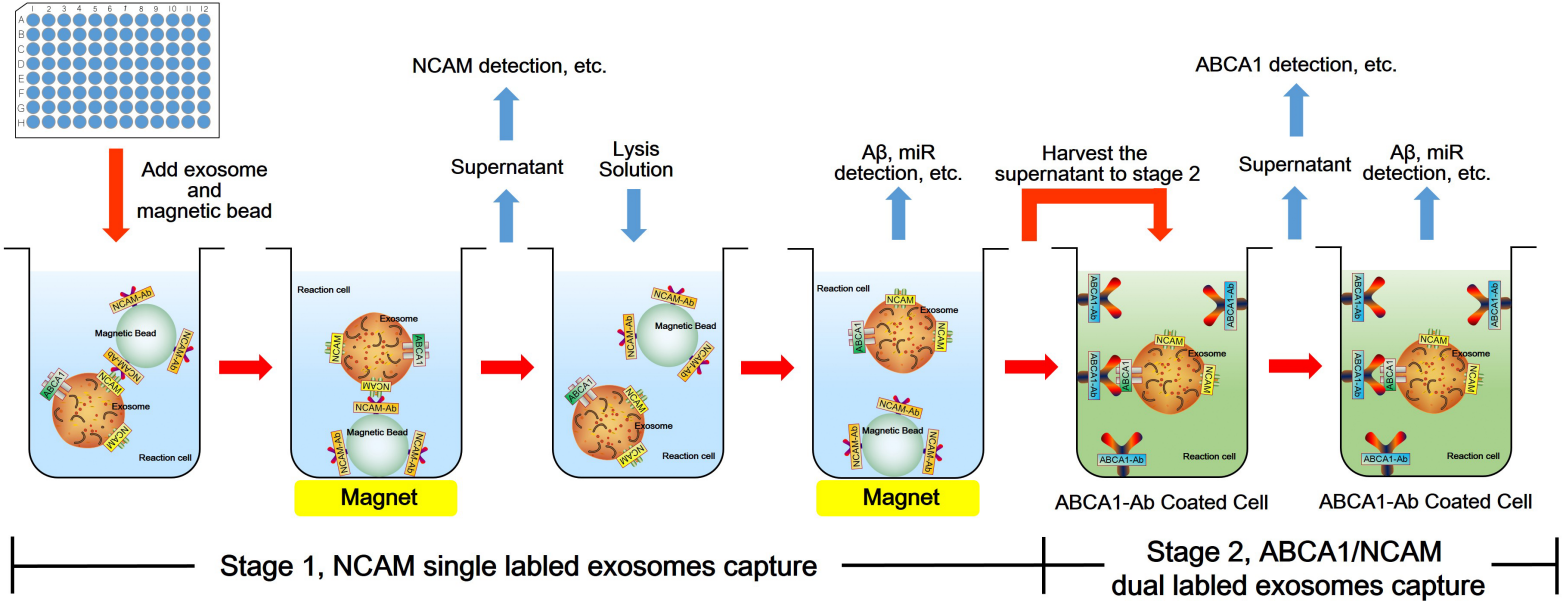
The procedures for collecting neuron‐derived exosomes from plasma. Stage 1 was used for NCAM single‐labeled exosomes capture. Stages 1 and 2 were used together for NCAM/ABCA1 dual‐labeled exosomes capture

Next, a specific subcomponent of neurogenic exosomes was captured. In brief, 200 μl supernatant from the previous step was added to the plate of a human ABCA1 ELISA kit (Abcam). After 30 min of incubation at 37°C, the plate was washed three times with the wash solution provided in the kit and then drained. Specific exosomes were adsorbed on the wall of the well for the next step. The extraction reagent was directly added to the well to extract protein or nucleic acid for subsequent detection, according to the following procedure (Figure 1).

### 2.5 Transmission electron microscopy

Exosomes adsorbed on the wall of the well were eluted using glycine–HCl (pH 3.0). The pH was adjusted to 7.0 using Tris–HCl (pH 8.6). The transmission electron microscopy (TEM) was performed according to a published protocol.^23^ Sections (65 nm) were stained with uranyl acetate and Reynold's lead citrate. The experiment was performed on JEM‐2100F TEM (JEOL,).

### 2.6 MiR purification and miR analysis

Total miR was purified using a magnetic bead method kit (Thermo Fisher Scientific) according to the manufacturer's protocol. Total RNA yields were about 20 ng/ml and 50 ng/ml in CSF and plasma, respectively, as assessed by Quant‐iT^TM^ RiboGreen^TM^ RNA reagent (Invitrogen). Total miR in blood cells was extracted using a column‐based method kit (Thermo Fisher Scientific). MiRs were reverse transcribed into cDNA with 10 μl reaction volume, followed by TaqMan qPCR according to the manufacturer's protocol (Thermo Fisher Scientific). Briefly, a 20 μl PCR reaction contained 2 μl cDNA, 300 nM TaqMan probe, 300 nM sense primer, and 300 nM antisense primer. Cycling parameters were 95°C for 8 min, followed by 35 cycles of 95°C for 15 s and 60°C for 1 min (Roche Light Cycler 480,). For body fluid samples, miRNeasy Serum/Plasma Spike‐In Control (*C*. *Elegans* miR‐39 miR mimic, Qiagen,) served as control. For cell samples, U6 snRNA (RNU6B, Qiagen) served as an endogenous control. The relative levels of miRs were calculated using the 2^−ΔΔCt^ method.^24^

### 2.7 Western blot analysis

Western blot analysis was performed to detect exosomal biomarkers, Alix, NCAM, and ABCA1 using monoclonal antihuman antibodies (Santa Cruz,). The dilutions of the primary/secondary antibodies of Alix and L1CAM were 1:1000/1:400 and 1:500/1:300, respectively. The captured exosomes and the corresponding supernatant in all steps were tested for the above three proteins.

### 2.8 ELISA

The Aβ42, Aβ40, T‐tau, P‐T181‐tau, and NfL (Abcam) ELISA tests were performed following the instructions of the kits. CD81 content was determined to normalize the exosome content. The mean value of CD81 level in each group was set to 1.00, and the relative value for each sample was used to normalize the recovery.^14^

### 2.9 Statistical analysis

Statistical analysis was performed using SPSS 18.0 for Windows (SPSS, Inc.,). The normality of quantitative data was tested using the Shapiro‐Wilk method. Normally distributed data were expressed as the mean ± standard deviation. The differences between groups were assessed using the *t*‐test or one‐way ANOVA. Rates were compared using the chi‐square (χ^2^) test. Correlations were determined by computing the Spearman Rank Correlation. The Delong test was used to compare the performance of two ROC curves. A binary logistic regression model was used to calculate the predicted values, where age, gender, and APOE status were used as covariates. A *p* value <0.05 was considered statistically significant.

## 3 RESULTS

### 3.1 Participant characteristics

Table 1 depicts the characteristics of participants. No significant differences were observed in the age and gender among the SCD, aMCI, AD, VaD, and control groups in both the discovery stage and the validation stage. The percentages of APOE ε4, Mini‐Mental State Examination, and Clinical Dementia Rating scores were significantly different between aMCI patients and controls, AD patients and controls, VaD patients and controls, as well as AD and aMCI patients (all *p* < 0.05).

**TABLE 1:** Demographics and clinical characteristics of subjects in this study

Characteristic	Total	Discovery Stage (*n* = 195)					Verification Stage (*n* = 197)				
		Control	VaD	SCD	aMCI	AD	Control	VaD	SCD	aMCI	AD
No. of subjects	392	30	30	45	50	40	30	30	47	45	45
Age, mean (SD), years	65.9 (6.6)	65.2 (6.5)	65.7 (6.1)	66.1 (5.9)	65.5 (7.1)	66.3 (6.5)	65.3 (6.1)	66.5 (5.6)	65.8 (5.5)	66.1 (7.1)	65.7 (6.6)
Males, No. (%)	190 (48.5)	15 (50.0)	15 (50.0)	20 (44.4)	25 (50.0)	20 (50.0)	15 (50.0)	15 (50.0)	21 (44.7)	22 (48.9)	22 (48.9)
Education years, mean (SD)	9.3 (1.5)	9.3 (1.4)	9.9 (1.5)	9.1 (1.5)	9.5 (1.4)	9.9 (1.4)	9.3 (1.4)	9.3 (1.5)	9.5 (1.4)	9.2 (1.3)	9.0 (1.4)
Body Mass Index, mean (SD)	25.9 (2.1)	25.3 (1.8)	25.9 (1.9)	26.2 (2.1)	25.8 (2.3)	26.0 (2.2)	25.6 (2.1)	26.3 (2.1)	25.7 (1.5)	26.0 (1.8)	25.7 (2.1)
ApoE ε4 positive, No. (%)	58 (14.8)	6 (20.0)	5 (16.7)	6 (13.3)	5 (10.0)	7 (17.5)	5 (16.7)	3 (10.0)	8 (17.0)*	6 (13.3)	7 (15.6)
MMSE score, mean (SD)	25.0 (1.9)	29.5 (1.3)	25.2 (0.6)*	28.6 (1.1)	26.1 (0.6)*^†^	20.9 (2.9)*^§^	29.7 (1.4)	24.9 (0.9)*	28.5 (1.0)	25.8 (0.5)*^†^	20.3 (2.6)*^§^
CDR score of 0/0.5/1.0/2.0, No.	206/118/46/22	29/1/0/0	9/19/2/0*	43/2/0/0	22/28/0/0*^†^	0/11/19/10*^§^	30/0/0/0	8/17/5/0*	44/3/0/0	21/24/0/0*^†^	0/13/20/12*^§^
Homocysteine, mean (SD), μmol/L	13.5 (3.5)	8.2 (2.1)	10.5 (3.6)*	16.7 (4.8)*	15.9 (3.9)*^†^	16.8 (3.2)*^§^	9.2 (2.3)	11.8 (3.0)*	16.5 (3.9)*	17.3 (4.6)*^†^	16.1 (3.7)*^§^
Hypertension treatment, No. (%)	64 (16.3)	5 (16.7)	6 (20.0)	7 (15.6)	7 (14.0)	5 (12.5)	6 (20.0)	5 (16.7)	7 (14.9)	8 (17.8)	8 (17.8)
Statin treatment, No. (%)	86 (21.9)	6 (20.0)	15 (50.0)*	5 (11.1)	8 (16.0)	7 (17.5)	5 (16.7)	21 (70.0)*	8 (17.0)	6 (13.3)	5 (11.1)

Abbreviations: APOE ε4, apolipoprotein ε4; CDR, clinical dementia rating; MMSE, Mini‐Mental State Examination.

*p < 0.05 compared to controls.

†*p* < 0.05 compared to SCD.

§<0.05 compared to aMCI.

### 3.2 Identification of exosomes

**FIGURE 2:**
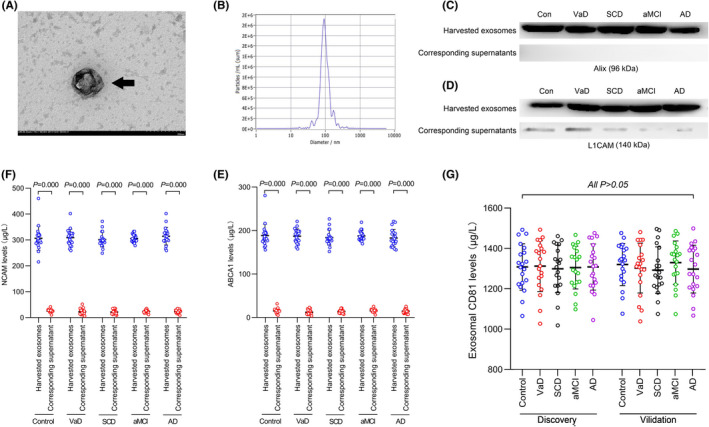
Confirmation of exosome collection by TEM and western blot and normalization of exosome content. A: Representative TEM image showing neuron‐derived exosomes (black arrow) that were successfully collected. B: Laser scattering microscopy shows that the size of the extract was consistent with the general size of exosomes. C–D: Representative western blot images show that the exosomal marker Alix (C) and L1CAM (D) were highly expressed in exosome samples but not detected or very low in corresponding supernatants. E–F: ELISA in controls, VaD, SCD, aMCI, and AD patients in the discovery stage shows that the NCAM (E) and ABCA1 (F) concentrations in captured exosomes were increased by approximately 10‐fold compared with those in corresponding supernatants. G: CD81 was measured to normalize the exosome content. No difference was detected between each group. *n* = 20 in each group. The *p* values are shown in the figure

The results of TEM and particle size analysis showed that the shape and size of the extract were consistent with the characteristics of exosomes (Figure 2A,B). Western blot results showed that Alix and L1CAM were highly expressed in stage 1 immune capture products but undetectable in supernatants (Figure 2C,D). The results of ELISA showed that in stage 1 and stage 2 immune captures, the NCAM and ABCA1 levels were higher than those of the corresponding supernatant (all *p* < 0.05) (Figure 2E,F).

The concentration of exosomes in all specimens was standardized using CD81 test results before testing other proteins. No significant difference was observed in CD81 concentrations among the groups (all *p* < 0.05) (Figure 2G).

**FIGURE 3:**
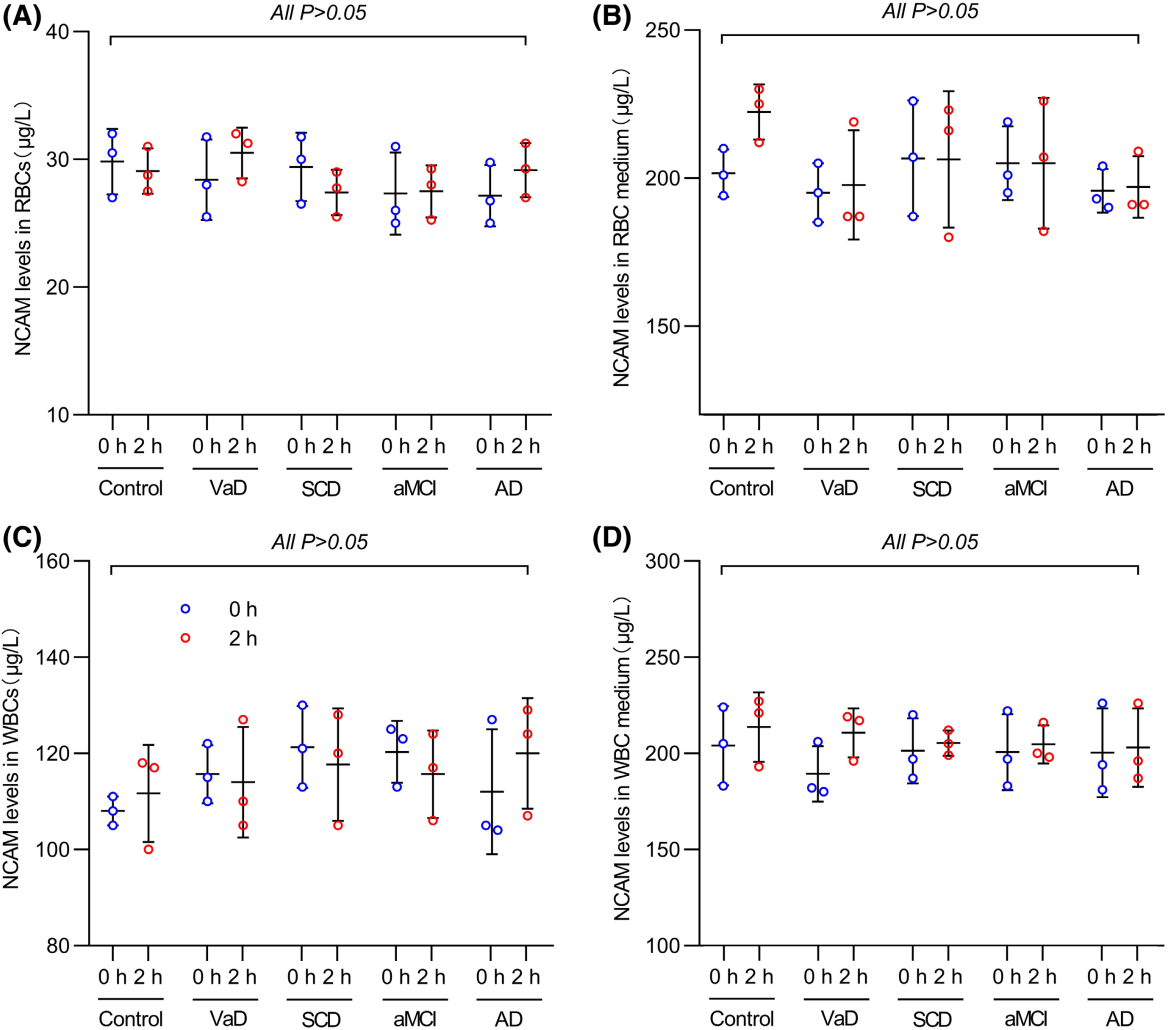
WBC and RBC NCAM secretion experiment results. A–B: After 2 h of incubation, NCAM levels in WBCs and RBCs did not decrease significantly (all *p* > 0.05). C–D: After 2 h of cultivation, the NCAM levels in WBC and RBC medium did not increase significantly (all *p* > 0.05). *n* = 3 in each group

Importantly, after 2 h of cultivation, the NCAM levels in WBCs and RBCs or the corresponding medium did not change significantly (all *p* > 0.05) (Figure 3). These results suggest that the content of NCAM in RBCs and WBCs was low (~30 μg/L) and that RBCs and WBCs barely release NCAM into the medium. Thus, specimen storage and pre‐processing may not affect the test results.

### 3.3 The Aβ42, Aβ_42/40_, T‐tau, P‐T181‐tau, NfL, and miR‐384 levels in NCAM single‐labeled exosomes of plasma

**FIGURE 4:**
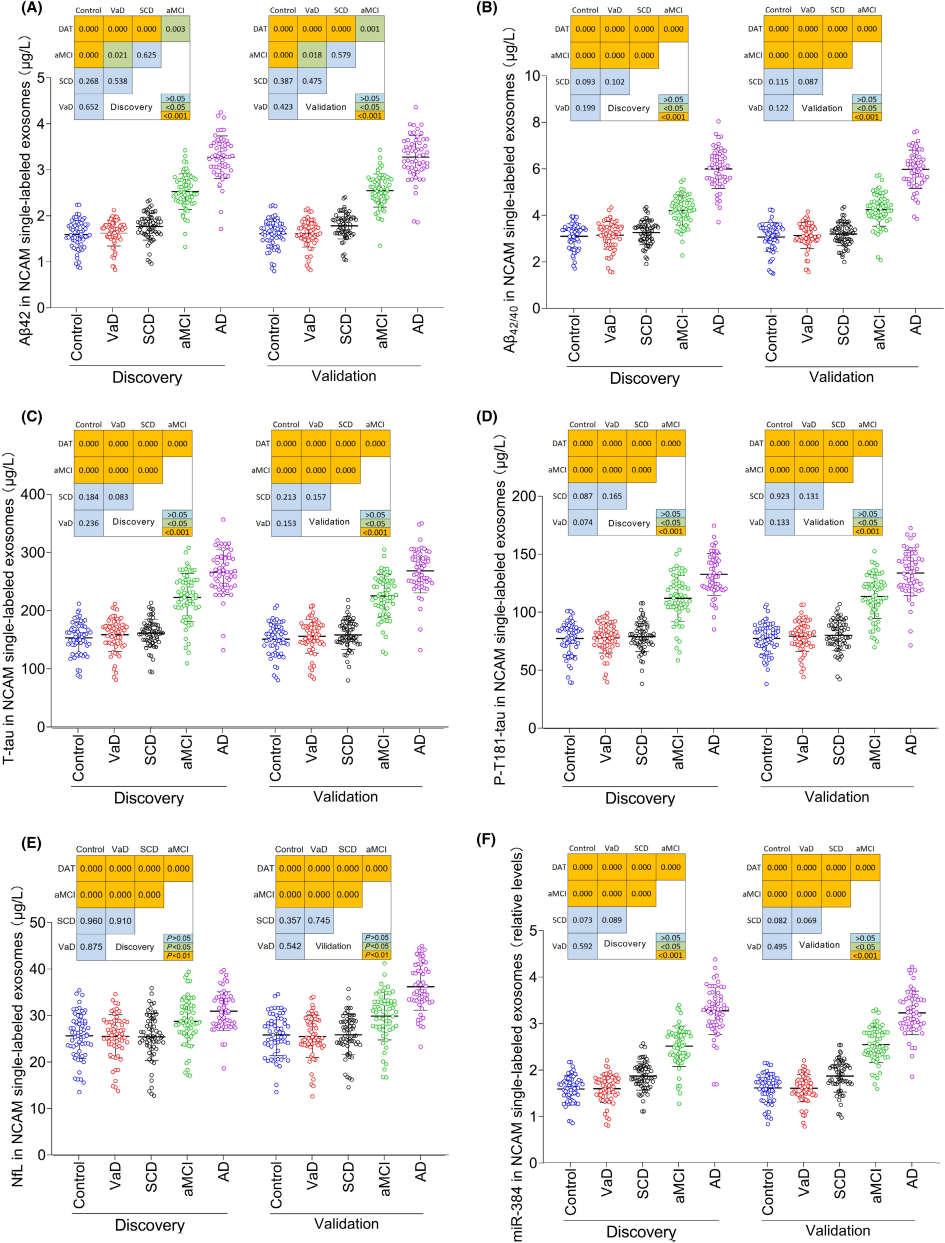
The Aβ42 (A), Aβ_42/40_ (B), T‐tau (C), P‐T181‐tau (D), NfL (E), and miR‐384 (F) levels in NCAM single‐labeled exosomes of plasma. In the discovery stage, *n* = 30 (Control), 30 (VaD), 45 (SCD), 50 (aMCI), and 40 (AD). In the validation stage, *n* = 30 (Control), 30 (VaD), 47 (SCD), 45 (aMCI), and 45 (AD). The *P* values are shown in the figure

We first used the specimens from the discovery stage to detect the NCAM‐labeled exosomal proteins extracted in the first stage of the exosome extraction experiment. Exosomes of this component are generally considered neuron‐derived exosomes. The results showed that the plasma exosomal Aβ42, Aβ_42/40_, Tau, and P‐T181‐tau levels of the aMCI and AD groups were significantly higher than those of the SCD group, control group, and VaD group, and those of the AD group were higher than those of the aMCI group. The plasma exosomal Aβ42, Tau, and P‐T181‐tau levels of the SCD group were slightly higher than those of the control group and the VaD group, but no significant difference was observed. The plasma exosomal NfL levels of the aMCI and AD groups were significantly higher than those of the SCD group, control group, and VaD group, with that of the AD group higher than that of the aMCI group. The plasma exosomal NfL level of the SCD group was slightly higher than that of the control group or the VaD group, but no significant difference was observed. The plasma exosomal miR‐384 levels of the aMCI and AD groups were significantly higher than those of the SCD, control, and VaD groups, and that of the AD group was higher than that of the aMCI group. The plasma exosomal miR‐384 level of the SCD group was slightly higher than that of the control group or the VaD group, but there was no significant difference. The results of the verification stage were consistent with those of the discovery stage (Figure 4).

### 3.4 The Aβ42, Aβ_42/40_, T‐tau, P‐T181‐tau, NfL, and miR‐384 levels in NCAM/ABCA1 dual‐labeled exosomes in blood and CSF

We first used the specimens from the discovery stage to detect the NCAM/ABCA1 dual‐labeled exosomal proteins extracted in the second stage of the exosome extraction experiment. The results showed that the plasma exosomal Aβ_42/40_, Tau, and P‐T181‐tau levels of the aMCI and AD groups were significantly higher than those of the SCD group, control group, and VaD group, and those of the AD groups were higher than those of the aMCI group. The plasma exosomal Aβ_42/40_, Tau, and P‐T181‐tau levels of the SCD group were slightly higher than those of the control group and the VaD group, but there was no significant difference. No significant difference was observed in exosomal Aβ40 levels among the groups, and only that of the AD group decreased slightly. The plasma exosomal NfL levels of the aMCI and AD groups were significantly higher than those of the SCD group, control group, and VaD group, and that of the AD group was higher than that of the aMCI group. The plasma exosomal NfL level of the SCD group was slightly higher than that of the control group or the VaD group, but there was no significant difference. The results of the verification stage were consistent with those the discovery stage. In all disease groups and control groups, the Aβ42 and Aβ_42/40_ levels in NCAM/ABCA1 dual‐labeled exosomes were higher than those of NCAM single‐labeled exosomes, as normalized to CD81.

**FIGURE 5:**
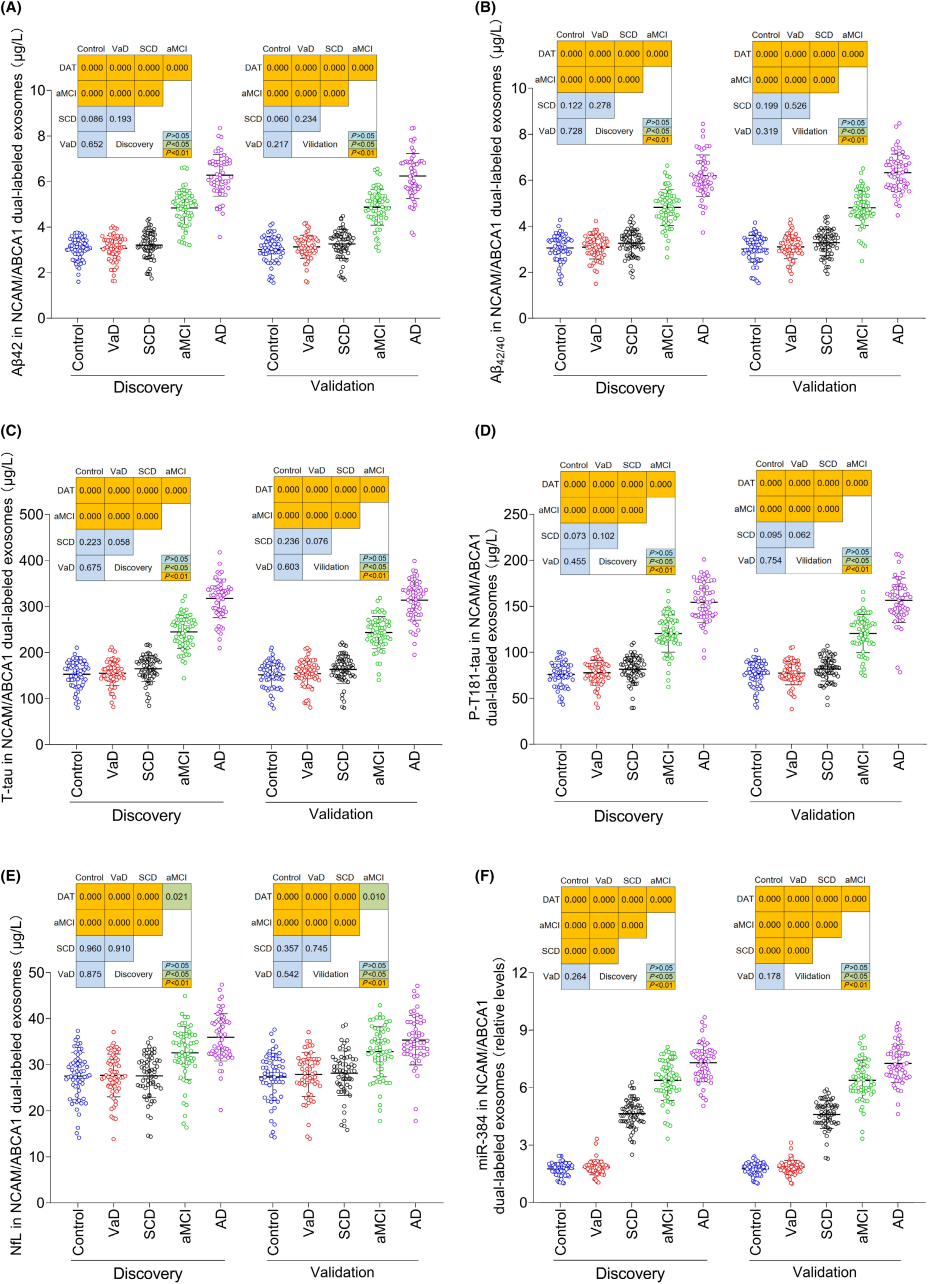
The Aβ42 (A), Aβ_42/40_ (B), T‐tau (C), p‐T181‐tau (D), NfL (E), and miR‐384 (F) levels in NCAM/ABCA1 dual‐labeled exosomes of plasma. In the discovery stage, *n* = 30 (Control), 30 (VaD), 45 (SCD), 50 (aMCI), and 40 (AD). In the validation stage, *n* = 30 (Control), 30 (VaD), 47 (SCD), 45 (aMCI), and 45 (AD). The *P* values are shown in the figure

The Aβ42 and miR‐384 levels in plasma NCAM/ABCA1 dual‐labeled exosomes of the SCD, aMCI, and AD groups were significantly higher than those of the control and VaD groups. Those of the AD group were higher than those the aMCI group, and those of the aMCI group were higher than those of the SCD group (Figure 5).

**FIGURE 6:**
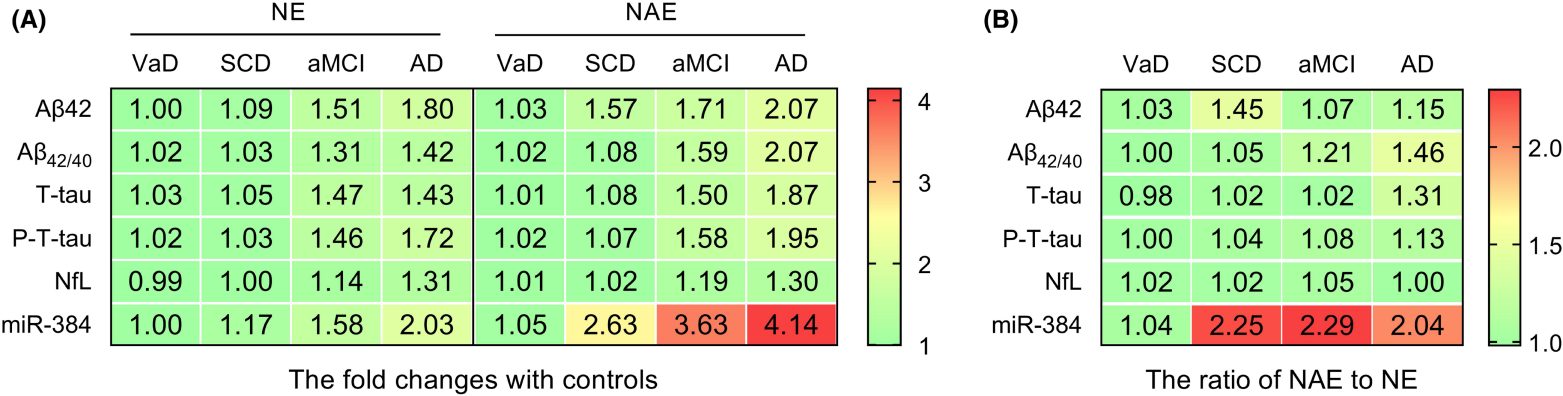
The fold changes of biomarkers. A shows the ratio of each biomarker relative to the control group in NCAM single‐labeled exosomes (NE) and NCAM/ABCA1 dual‐labeled exosomes (NAE) from VaD, SCD, aMCI, and AD. B shows the ratio of NAE to NE

Of interest, the increased ratio of NCAM/ABCA1 double‐labeled exosomal Aβ42 and miR‐384 in all disease groups was higher than that of T‐tau, P‐T181‐tau, and NfL (Figure 6).

### 3.5 Correlation analysis between CSF and plasma exosomal biomarkers

**FIGURE 7:**
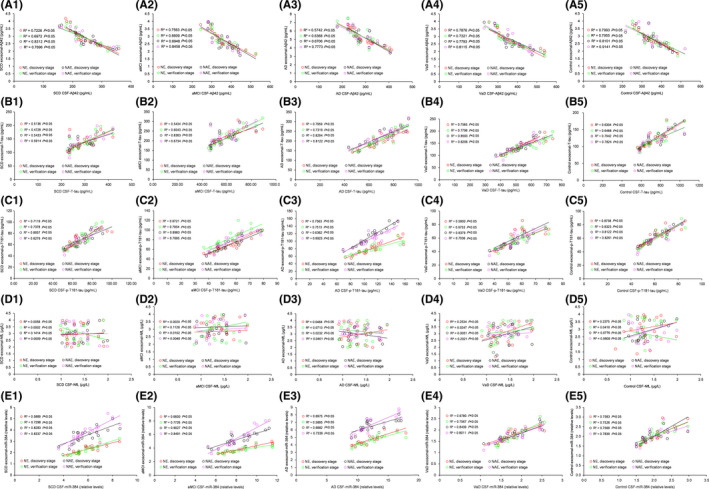
The exosomal biomarker levels are significantly correlated with the CSF biomarker levels. A, C, and E show robust correlations of Aβ42 (A), P‐T181‐tau (C), and miR‐384 (E) from exosomes and CSF in SCD (1), aMCI (2), AD (3), VaD (4), and controls (5) (all *p* < 0.05). B shows significant correlations of T‐tau from exosomes and CSF in SCD (1), aMCI (2), AD (3), VaD (4), and controls (5) (all *p* < 0.05). C shows no correlation of NfL from exosomes and CSF in SCD (1), aMCI (2), AD (3), VaD (4), and controls (5) (all *p* > 0.05). *n* = 20 in each group. NE =NCAM single‐labeled exosomes, NAE =NCAM/ABCA1 dual‐labeled exosomes

We conducted a correlation analysis of exosomal and CSF biomarkers and found that the Aβ42, P‐T181‐tau, and miR‐384 levels in neuron‐derived exosomes were highly correlated with their levels in CSF in all groups (all *p* < 0.05), except for the NfL level (all *p* > 0.05). There, correlations were also observed in NCAM/ABCA1 dual‐labeled exosomes, with higher correlation coefficients (Figure 7).

### 3.6 Diagnostic power analysis of plasma exosomal biomarkers

**FIGURE 8:**
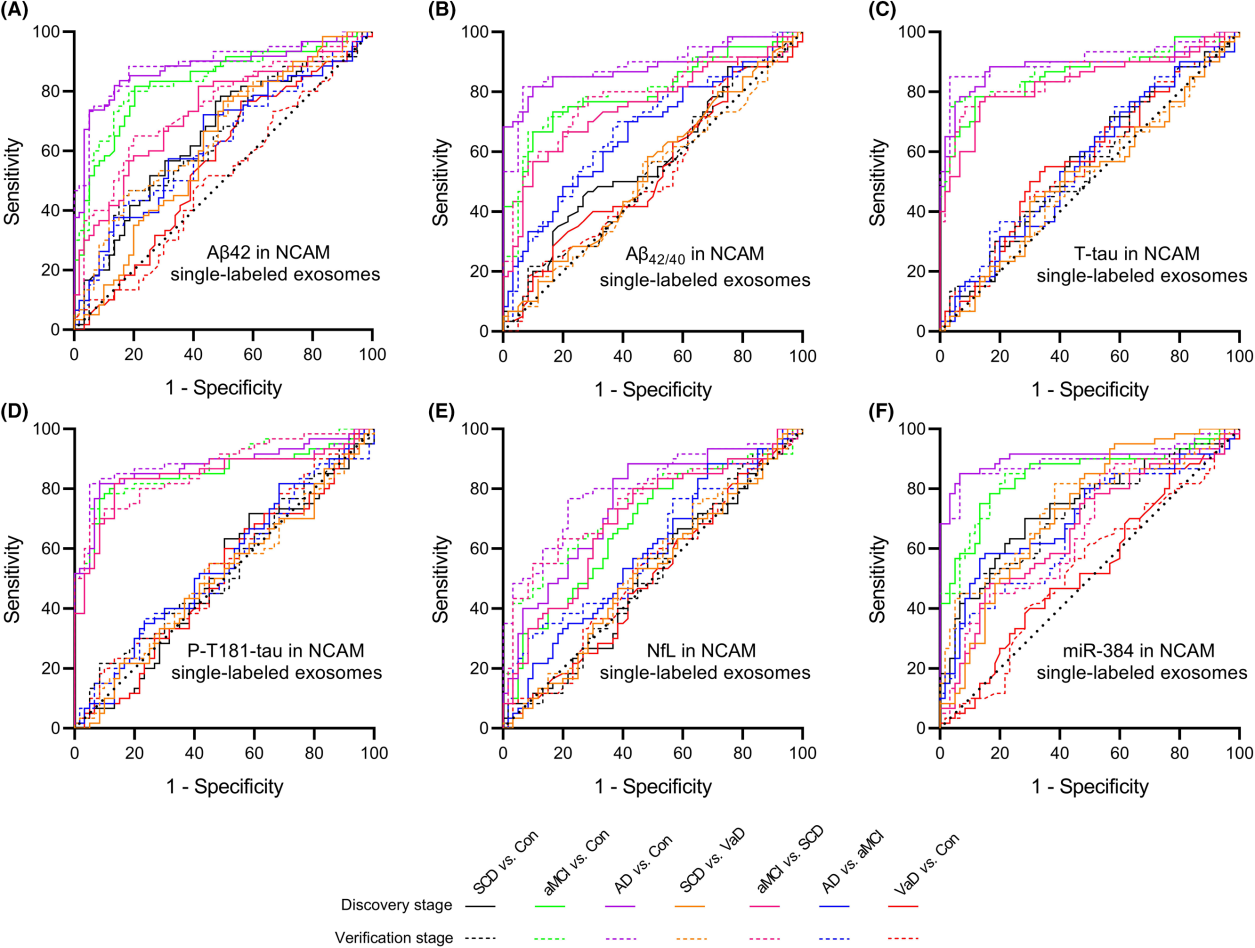
Diagnostic power analysis of plasma exosomal biomarkers in NCAM single‐labeled exosomes of plasma. A–F show multiple ROC analyses of Aβ42 (A), Aβ_42/40_ (B), T‐tau (C), P‐T181‐tau (D), NfL (E), and miR‐384 (F) in NCAM single‐labeled exosomes of plasma in SCD, aMCI, AD, VaD, and controls. In the discovery stage, *n* = 30 (Control), 30 (VaD), 45 (SCD), 50 (aMCI), and 40 (AD). In the validation stage, *n* = 30 (Control), 30 (VaD), 47 (SCD), 45 (aMCI), and 45 (AD). The *P* values are shown in the figure. Con =control

**FIGURE 9:**
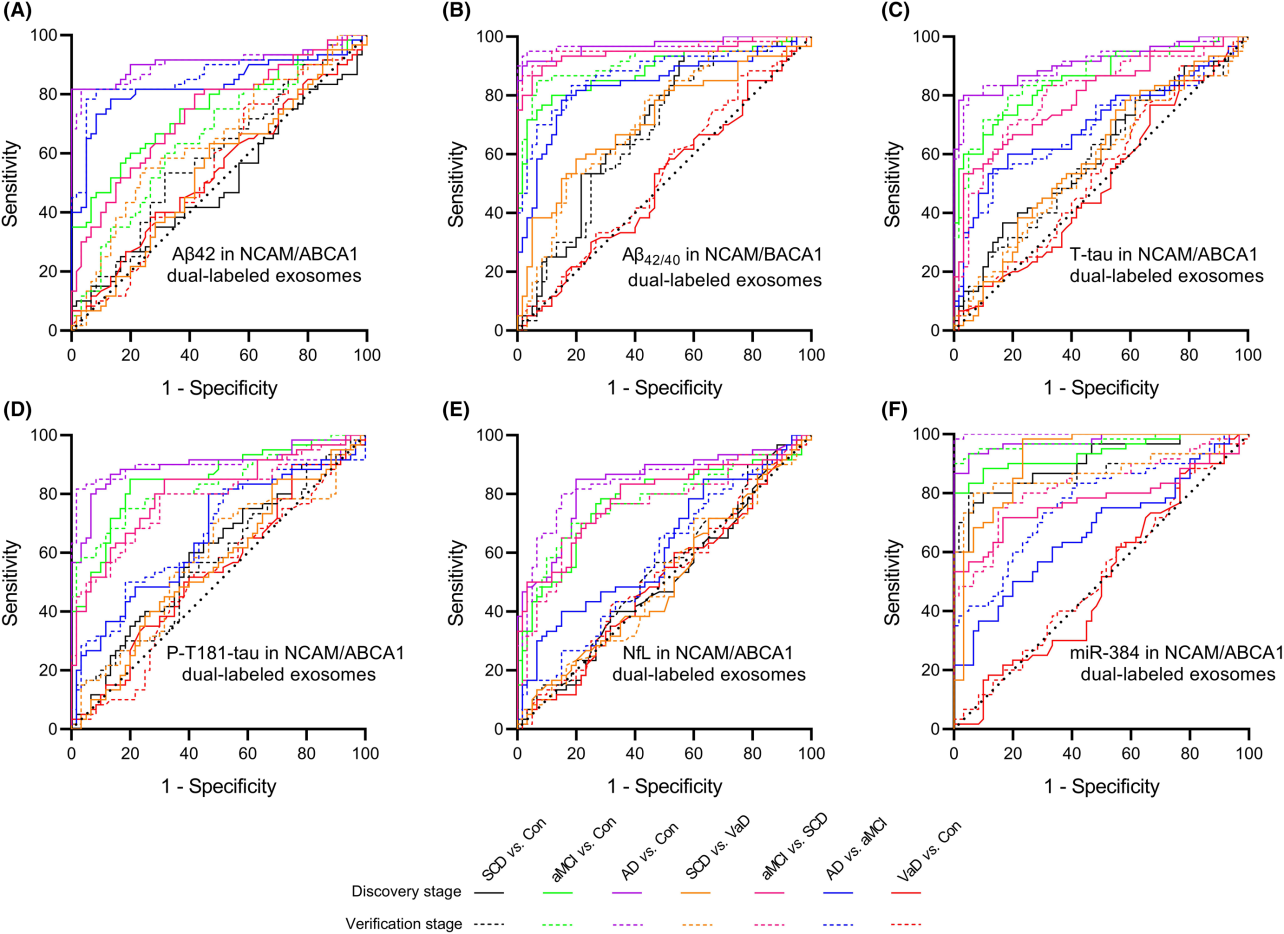
Diagnostic power analysis of biomarkers in NCAM/ABCA1 dual‐labeled exosomes of plasma. A–F show multiple ROC analyses of Aβ42 (A), Aβ_42/40_ (B), T‐tau (C), P‐T181‐tau (D), NfL (E), and miR‐384 (F) in NCAM/ABCA1 dual‐labeled exosomes of plasma in SCD, aMCI, AD, VaD, and controls. In the discovery stage, *n* = 30 (Control), 30 (VaD), 45 (SCD), 50 (aMCI), 40 (AD). In the validation stage, *n* = 30 (Control), 30 (VaD), 47 (SCD), 45 (aMCI), 45 (AD). The *P* values are shown in the figure. Con =control

We used the ROC curve to analyze the diagnostic performance of Aβ42, Aβ_42/40_, T‐tau, P‐T181‐tau, NfL, and miR‐384 in plasma NCAM single‐labeled (Figure 8, Table 2) and NCAM/ABCA1 dual‐labeled (Figure 9, Table 3) exosomes. Plasma NCAM/ABCA1 dual‐labeled exosomal Aβ_42/40_ and miR‐384 showed a good diagnostic performance. For Aβ_42/40_, the AUCs for the SCD/controls, aMCI/controls and AD/controls comparisons were 0.698 (*p* = 0.000) and 0.884 (*p* = 0.000), 0.969 (*p* = 0.000), respectively; the AUCs for the aMCI/SCD and AD/aMCI were 0.828 (*p* = 0.000) and 0.806 (*p* = 0.000), respectively. For miR‐384, the AUCs for the SCD/controls, AD/controls and aMCI/controls comparisons were 0.876 (*p* = 0.000) and 0.906 (*p* = 0.000), 0.915 (*p* = 0.000), respectively; the AUCs for the SCD/aMCI and aMCI/AD were 0.778 (*p* = 0.000) and 0.670 (*p* = 0.000), respectively. The results of DeLong test showed that the AUC of NCAM/ABCA1 dual‐labeled exosomal Aβ_42/40_ for diagnosis of SCD was higher than that of Aβ42 (*p* = 0.032), T‐tau (*p* = 0.001), and P‐T181‐tau (*p* = 0.025); the AUC of NCAM/ABCA1 dual‐labeled exosomal miR‐384 for diagnosis of SCD was higher than that of Aβ42 (*p* = 0.027), Aβ_42/40_ (*p* = 0.001)_,_ T‐tau (*p* = 0.001), P‐T181‐tau (*p* = 0.008), and NfL (*p* = 0.000). These data indicate that plasma NCAM/ABCA1 dual‐labeled exosomal Aβ_42/40_ and miR‐384 had potential advantages in the diagnosis of SCD.

**TABLE 2:** The ROC curve analysis results of NCAM single‐labeled exosomal biomarkers

	Stage	VaD *vs*. Con		SCD *vs*. Con		aMCI *vs*. Con		AD *vs*. Con		SCD *vs*. VaD		aMCI *vs*. SCD		AD *vs*. aMCI	
		AUC	*p*	AUC	*p*	AUC	*p*	AUC	*p*	AUC	*p*	AUC	*p*	AUC	*p*
Aβ42	Discovery	0.557	0.285	0.651	0.004	0.834	0.000	0.879	0.000	0.618	0.026	0.734	0.000	0.617	0.027
	Verification	0.504	0.939	0.641	0.051	0.841	0.000	0.890	0.000	0.657	0.003	0.747	0.000	0.637	0.051
Aβ_42/40_	Discovery	0.534	0.517	0.571	0.181	0.802	0.000	0.887	0.000	0.532	0.541	0.754	0.000	0.666	0.002
	Verification	0.529	0.583	0.537	0.482	0.802	0.000	0.891	0.000	0.505	0.919	0.783	0.000	0.678	0.001
T‐tau	Discovery	0.573	0.168	0.566	0.216	0.859	0.000	0.885	0.000	0.514	0.797	0.833	0.000	0.558	0.270
	Verification	0.565	0.217	0.558	0.276	0.876	0.000	0.908	0.000	0.509	0.862	0.852	0.000	0.576	0.149
P‐T181‐tau	Discovery	0.500	0.994	0.502	0.996	0.857	0.000	0.878	0.000	0.503	0.963	0.846	0.000	0.546	0.389
	Verification	0.541	0.440	0.533	0.537	0.879	0.000	0.879	0.000	0.509	0.858	0.868	0.000	0.531	0.564
NfL	Discovery	0.501	0.985	0.508	0.875	0.676	0.001	0.751	0.000	0.513	0.813	0.693	0.000	0.586	0.103
	Verification	0.522	0.673	0.508	0.879	0.742	0.000	0.803	0.000	0.529	0.582	0.746	0.000	0.599	0.062
miR−384	Discovery	0.527	0.616	0.740	0.000	0.846	0.000	0.884	0.000	0.726	0.000	0.663	0.002	0.712	0.000
	Verification	0.528	0.598	0.717	0.000	0.853	0.000	0.808	0.000	0.738	0.000	0.661	0.002	0.669	0.001

Abbreviation: Con, Control.

**TABLE 3:** The ROC curve analysis results of NCAM/ABCA1 dual‐labeled exosomal biomarkers

	Stage	VaD *vs*. Con		SCD *vs*. Con		aMCI *vs*. Con		AD *vs*. Con		SCD *vs*. VaD		aMCI *vs*. SCD		AD *vs*. aMCI	
		AUC	*p*	AUC	*p*	AUC	*p*	AUC	*p*	AUC	*p*	AUC	*p*	AUC	*p*
Aβ42	Discovery	0.538	0.477	0.500	0.999	0.749	0.000	0.910	0.000	0.538	0.472	0.735	0.000	0.802	0.000
	Verification	0.549	0.353	0.590	0.090	0.653	0.004	0.904	0.000	0.644	0.006	0.735	0.000	0.811	0.000
Aβ_42/40_	Discovery	0.506	0.916	0.698	0.000	0.884	0.000	0.969	0.000	0.715	0.000	0.828	0.000	0.806	0.000
	Verification	0.533	0.537	0.671	0.001	0.885	0.000	0.973	0.000	0.717	0.000	0.832	0.000	0.784	0.000
T‐tau	Discovery	0.503	0.962	0.598	0.064	0.856	0.000	0.910	0.000	0.591	0.087	0.796	0.000	0.703	0.000
	Verification	0.536	0.493	0.588	0.095	0.861	0.000	0.908	0.000	0.566	0.214	0.806	0.000	0.691	0.000
P‐T181‐tau	Discovery	0.540	0.445	0.591	0.084	0.849	0.000	0.900	0.000	0.553	0.319	0.807	0.000	0.662	0.002
	Verification	0.511	0.842	0.568	0.198	0.834	0.000	0.898	0.000	0.576	0.153	0.785	0.000	0.668	0.002
NfL	Discovery	0.501	0.979	0.502	0.971	0.706	0.000	0.838	0.000	0.503	0.950	0.787	0.000	0.622	0.021
	Verification	0.521	0.698	0.537	0.490	0.696	0.000	0.843	0.000	0.506	0.904	0.757	0.000	0.578	0.139
miR−384	Discovery	0.502	0.973	0.862	0.000	0.915	0.000	0.906	0.000	0.804	0.000	0.778	0.000	0.670	0.001
	Verification	0.517	0.743	0.876	0.000	0.914	0.000	0.909	0.000	0.802	0.000	0.829	0.000	0.768	0.000

Abbreviation: Con, Control.

### 3.7 Analysis of diagnostic efficacy of combination biomarkers

**FIGURE 10:**
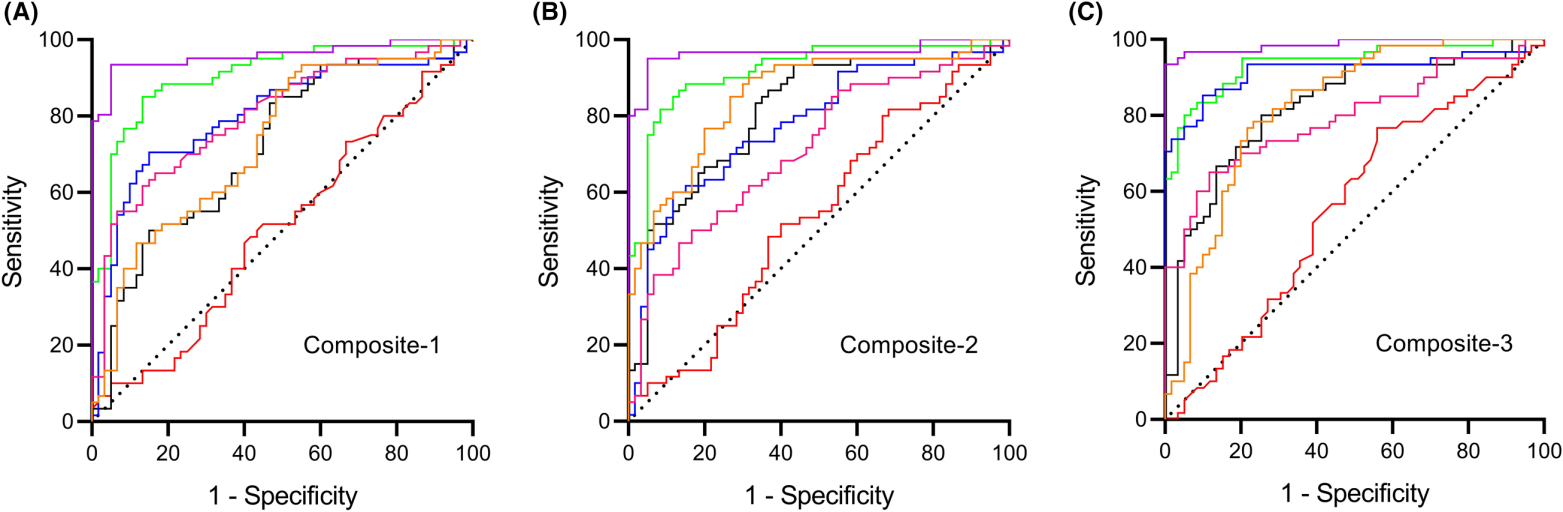
Higher performance of the combined biomarkers. The data of the discovery and validation stages were integrated for analysis. The performances of combined analysis of Aβ42, Aβ_42/40_, T‐tau, P‐T181‐tau, NfL, and miR‐384 in plasma NCAM single‐labeled exosomes, plasma NCAM/ABCA1 dual‐labeled exosomes or CSF as composite‐1 (A), composite‐2 (B), and composite‐3 (C), respectively. *n* = 60 (Control), 60 (VaD), 92 (SCD), 95 (aMCI), 85 (AD)

Further, we used logistic regression analysis and ROC to evaluate the diagnostic efficacy of the combination of the above‐mentioned biomarkers for AD (Figure 10, Table 4). The biomarkers in plasma NCAM single‐labeled exosomes, plasma NCAM/ABCA1 dual‐labeled exosomes or CSF were defined as composite‐1, composite‐2, and composite‐3, respectively. We compared the AUCs between the discovery and validation data sets and found no differences in the AUCs of each biomarker. Thus, the data of the two stages were integrated for analysis. The three combinations achieved similar diagnostic performance. These data suggest that the combination of exosomal biomarkers had slightly higher diagnostic efficiency than the individual biomarkers and that the exosomal biomarkers had the same diagnostic power as the CSF biomarkers.

**TABLE 4:** The ROC curve analysis results of combination biomarkers

Composite	VaD *vs*. Con		SCD *vs*. Con		aMCI *vs*. Con		AD *vs*. Con		SCD *vs*. VaD		aMCI *vs*. SCD		AD *vs*. aMCI	
	AUC	*p*	AUC	*p*	AUC	*p*	AUC	*p*	AUC	*p*	AUC	*p*	AUC	*p*
Composite−1	0.504	0.939	0.718	0.000	0.855	0.000	0.919	0.000	0.730	0.000	0.800	0.000	0.832	0.000
Composite−2	0.539	0.466	0.810	0.000	0.879	0.000	0.915	0.000	0.854	0.000	0.711	0.000	0.878	0.000
Composite−3	0.557	0.285	0.823	0.000	0.908	0.000	0.927	0.000	0.821	0.000	0.814	0.000	0.919	0.000

Abbreviation: Con, Control

## 4 DISCUSSION

Exosomes are tiny vesicles actively secreted by cells, carrying materials and biological information transmitted by cells.^25^ In recent years, a variety of exosome capture techniques have been developed, such as microfluidic chip technology and immune capture.^14^, ^22^ The core principle of the immune capture method is based on the size of the exosomes (30–200 nm), which is comparable to the size of virus‐like particles and viruses (within 300 nm) that can be captured by antibodies.^26^ Based on the previous research, this study combined the magnetic bead method and the microtiter plate method to make it possible to harvest the exosomes with dual‐specific biomarkers for ideal AD diagnostic performance.

In 2015, a single‐center study has reported that peripheral blood exosomal Aβ42, T‐tau, and P‐T181‐tau can identify or predict AD up to 10 years before clinical onset.^22^ The characteristics of exosomal Aβ42, T‐tau, and P‐T181‐tau were different from that of NfL, which was mainly used for diagnosis and early warning of familial AD.^8^ Furthermore, the results of a multicenter study have confirmed the above conclusion. The peripheral blood and CSF test results of 298 subjects from 8 centers have shown that the NCAM single‐labeled Aβ42 and other indicators have a good correlation with CSF.^14^ Considering that the CSF Aβ42 level can reflect the degree of the amyloid plate and other pathological changes in brain tissue, the plasma exosomal Aβ42 level might also reflect the degree of the amyloid plate in brain tissue to a certain extent. Our findings further confirmed that plasma NCAM single‐labeled exosomal Aβ42, Aβ_42/40_, T‐tau, and P‐T181‐tau have promising applications in the diagnosis of aMCI and AD as well as the differential diagnosis of VaD, serving as a substitute for CSF markers for inspection and PET inspection.

BACE‐1 is a key enzyme in the occurrence and development of AD. Our previous study has shown that miR‐384 can downregulate the expression and activity of BACE‐1.^12^ However, with the development of AD, the miR‐384 level in neuronal cells continued to decrease. Further animal experiments and clinical studies have shown that with the progress of AD, the miR‐384 level in CSF and peripheral blood exosomes keep rising, suggesting that the decrease in neuronal cellular miR‐384 may be due to excessive miR‐384 being transported from neurons to CSF and peripheral blood.

The pathogenesis of AD is associated with multiple factors, including genetic alterations, amyloid plaque formation, tau protein deposition, and dysregulation of lipid metabolism. ABCA1 expressed in the neurons can transport cholesterol and other lipids from the neurons to the extracellular environment by consuming adenosine triphosphate.^27^ Apolipoprotein E4 (ApoE4) plays a critical role in amyloid production and tau protein phosphorylation in the neurons.^15^ ABCA1 is responsible for ApoE4 lipidation, acting as a component of the amyloid clearance channel. ABCA1 also transports soluble Aβ into BBB endothelial cells, contributing to Aβ degradation in endothelial cells or its release into the peripheral blood crossing BBB.^28^, ^29^ Therefore, decreased neuronal ABCA1 expression is involved in the occurrence and development of AD.^28^ As a transmembrane protein, ABCA1 may be loaded on the exosomal membrane during the budding of exosomes. Our recent study has shown that CSF and serum exosomal ABCA1 levels are remarkably elevated in AD patients compared with those in healthy controls,^26^ suggesting that exosomal transport of ABCA1 and miR‐384 are correlated during the development of AD. Therefore, we investigated ABCA1‐labeled exosomes in this study. Our results suggest that exosomal transport of ABCA1/miR‐384 from the neurons to CSF and peripheral blood contributes to abnormal amyloid deposition in the brain and subsequent development of AD.

The current study showed that the diagnostic efficacy of miR‐384 in peripheral blood NCAM single‐labeled exosomes for AD and aMCI was similar to that of Aβ42, but their diagnostic value for SCD was limited. However, miR‐384 in NCAM/ABCA1 dual‐labeled exosomes of peripheral blood not only had a better diagnostic performance for AD and aMCI but also had an acceptable diagnostic performance for SCD, which was not achieved by peripheral blood exosomal Aβ42.

In addition, no significant differences were observed in T‐tau, P‐T181‐tau, and NfL levels as well as their efficiencies for AD diagnosis and staging between NCAM single‐labeled and NCAM/ABCA1 double‐labeled exosomes, suggesting that there is no need to detect NCAM/ABCA1 dual‐labeled exosomal T‐tau, P‐T181‐tau, and NfL for diagnosis and staging of AD. These data suggest that blood neuron‐derived exosomes are ideal biomarker carriers for AD screening, especially for large‐scale population screening. We also noticed that a small proportion of the control group had relatively high levels of biomarkers. However, the statistical difference between the control group and the aMCI/AD group was significant, suggesting potential diagnostic values of the biomarkers. These biomarkers can be used in early detection and risk assessment of aMCI/AD, but the diagnosis of aMCI/AD requires other signs, symptoms, and tests.

In summary, this study, for the first time, established a method to sort specific surface marker exosomes using a two‐step method. We demonstrated that peripheral neuronal‐derived exosomal Aβ42, Aβ_42/40_, T‐tau, P‐T181‐tau, and miR‐384 may reflect AD pathological changes in the brain and therefore can diagnose AD. The detection of NCAM/ABCA1 dual‐labeled exosomal molecules showed a better potential diagnostic value for SCD than NCAM single‐labeled exosomes. However, these findings need further confirmation in longitudinal studies.

## CONFLICTS OF INTEREST

The authors declare that they have no conflicts of interest.

## Data Availability

The data that support the findings of this study are available from the corresponding author upon reasonable request.
